# Disulfidptosis-related signature predicts prognosis and characterizes the immune microenvironment in hepatocellular carcinoma

**DOI:** 10.1186/s12935-023-03188-y

**Published:** 2024-01-09

**Authors:** Jun Tang, Xintong Peng, Desheng Xiao, Shuang Liu, Yongguang Tao, Long Shu

**Affiliations:** 1 Key Laboratory of Carcinogenesis and Cancer Invasion (Ministry of Education), NHC Key Laboratory of Carcinogenesis (Central South University), Cancer Research Institute and School of Basic Medicine, Central South University, Changsha, 410078 Hunan China; 2 Hunan Key Laboratory of Cancer Metabolism, Hunan Cancer Hospital and The Affiliated Cancer Hospital of Xiangya School of Medicine, Central South University, Changsha, 410078 Hunan China; 3 Department of Thoracic Surgery, Hunan Key Laboratory of Early Diagnosis and Precision Therapy in Lung Cancer, Second Xiangya Hospital, Central South University, Changsha, 410011 China; 4 Department of Pathology, Xiangya Hospital, Central South University, Changsha, 410008 Hunan China; 5 Department of Oncology, Institute of Medical Sciences, National Clinical Research Center for Geriatric Disorders, Xiangya Hospital, Central South University, Changsha, 410008 Hunan China

**Keywords:** Disulfidptosis, Hepatocellular carcinoma, Immune microenvironment, Prognostic model

## Abstract

**Background:**

Disulfidptosis is a type of programmed cell death caused by excessive cysteine-induced disulfide bond denaturation leading to actin collapse. Liver cancer has a poor prognosis and requires more effective intervention strategies. Currently, the prognostic and therapeutic value of disulfidptosis in liver cancer is not clear.

**Methods:**

We investigated the features of 16 disulfidptosis-related genes (DRGs) of HCC patients in the TCGA and classified the patients into two disulfidptosis pattern clusters by consensus clustering analysis. Then, we constructed a prognostic model using LASSO Cox regression. Next, the microenvironment and drug sensitivity were evaluated. Finally, we used qPCR and functional analysis to verify the reliability of hub DRGs.

**Results:**

Most of the DRGs showed significantly higher expression in cancer tissues than in adjacent tissues. Our prognostic model, the DRG score, can well predict the survival of HCC patients. There were significant differences in survival, features of the microenvironment, effects of immunotherapy, and drug sensitivity between the high- and low-DRG score groups. Ultimately, we demonstrated that a few hub DRGs have differential mRNA expression between liver cancer cells and normal cells and that the protective gene LCAT can inhibit liver cancer metastasis in vitro.

**Conclusion:**

We established a novel risk model based on DRG scores to predict HCC patient prognosis, drug sensitivity and immunotherapy efficacy, which provides new insight into the relationship between disulfidptosis and HCC and provides valuable assistance for the personalized treatment of HCC.

**Supplementary Information:**

The online version contains supplementary material available at 10.1186/s12935-023-03188-y.

## Introduction

Worldwide, hepatocellular carcinomas account for 80–90% of liver cancer cases, making it one of the most lethal tumors. The factors of liver cancer include aflatoxin, viral infections (HBV and HCV), metabolic alteration (alcoholic steatohepatitis, nonalcoholic steatohepatitis), obesity, smoking, etc. [1, 2]. Currently, the majority of patients with liver cancer receive their diagnosis at an advanced stage, and they typically have a dismal prognosis. In actuality, few patients are eligible for surgical procedures, and prolonged chemotherapy use increases the risk of toxicity or drug resistance. In addition, only 20% of patients are candidates for treatment with immune checkpoint inhibitors (ICPs). Therefore, it is imperative and urgent to continue researching possible targets for the treatment of liver cancer treatment [3, 4].

Disulfidptosis is a recently discovered form of cell death. It occurs in cells with high expression of SLC7A11, in which glucose depletion leads to a reduction in intracellular NADPH production. This results in the accumulation of cysteine, which causes abnormal accumulation of disulfides and ultimately leading to cell death through targeting of the actin cytoskeleton [5, 6]. Importantly, GLUT inhibitors can induce disulfidptosis in SLC7A11^high^ tumor cells, inhibiting tumor growth both in vitro and in vivo. The mechanism of disulfidptosis provides interesting potential targets for cancer treatment. Furthermore, metastatic cancer cells have more lamellipodia and invasive protrusions, suggesting that they may be more sensitive to disulfidptosis [7]. Targeting metabolic reprogramming is one of the strategies in cancer treatment, and the study of disulfidptosis, as a novel form of metabolism-related cell death, offers new insights for cancer therapy and clinical translation [8, 9].

Glucose metabolism is important for tumor growth and progression, and glycogen accumulation is an important event in inducing liver tumor initiation [10]. Furthermore, prohibiting tumor malignancy and reducing the incidence of hepatic carcinoma by removing excess glycogen from the body is an efficient approach. Notably, disulfidptosis is a novel type of programmed cell death (PCD), which is caused by actin cytoskeleton network collapse due to bond abnormalities abnormality under glucose conditions. Thus, disulfidptosis may be strongly associated with the retardation of the malignant progression of liver cancer. However, the molecular mechanism by which disulfidptosis suppresses liver cancer is currently unclear.

**Fig. 1:**
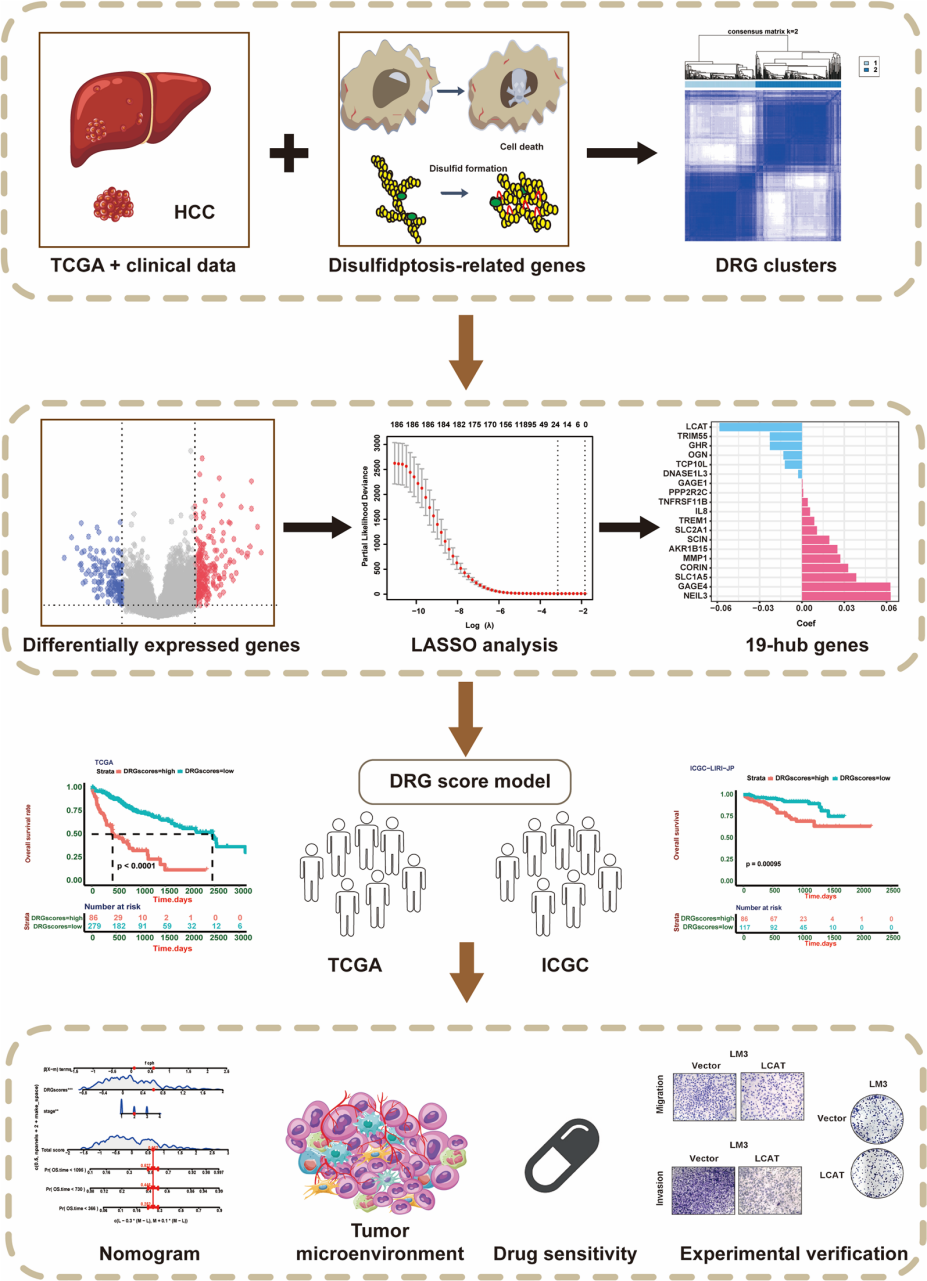
The overall experimental design and major steps of our study. We first investigated the expression of 16 disulfidptosis-related genes in HCC patients in the TCGA-LIHC cohort and categorized the HCC patients into two DRG clusters based on the expression of those genes. Subsequently, we selected DEGs with prognostic significance between DRG clusters for LASSO analysis. In total, 19 hub genes were selected for the construction of the DRG score model in the TCGA cohort, which was validated in an external ICGC cohort. Then, we developed a nomogram based on our model and assessed immune infiltration and drug sensitivity in HCC patients. Finally, through experimental validation, we confirmed the anticancer role of the hub gene LCAT in liver cancer and explored its potential association with disulfidptosis-related death. *DRG* Disulfidptosis-related gene, *HCC* hepatocellular carcinoma, *LASSO* least absolute shrinkage and selection operator, *DEG* differentially expressed gene

In this study, we explored the expression profiles of 16 DRGs and related features in HCC. A disulfidptosis-related risk model based on DRG scores was established to predict HCC patient features and prognosis. We also investigated the tumor microenvironment (TME) infiltration, drug sensitivity and immunotherapy response in different DRG score groups of HCC patients. Additionally, we revealed the role of LCAT induced disulfidptosis in the progression of liver cancer, providing new insight into liver cancer treatment via targeted disulfidptosis. The overall experimental design and major steps are could be shown in Fig. 1.

## Materials and methods

### Patient data source

The Log2 (RSEM + 1) normalized gene expression data, gene copy number variation data estimated using the GISTIC2 method, and corresponding clinical data of the TCGA-LIHC cohort were downloaded from UCSC Xena (https://xenabrowser.net/). The testing cohort International Cancer Genome Consortium (ICGC-LIRI-JP) for liver cancer was downloaded from the ICGC Data Portal (https://dcc.icgc.org/). We further included patients with OS time greater than 0, complete survival status information, and comprehensive clinical and pathological data. Table 1 presents the age, sex, pathological stage, and survival information of the included patients. The copy number variation of genes was visualized by the “RCircos” package.

**Table 1:** Clinical characteristics of HCC patients from TCGA and ICGC database

	TCGA	ICGC	Overall
Age (years)			
Mean (SD)	59.6 (13.4)	67.0 (10.2)	62.3 (12.8)
Median	61.0	69.0	64.0
Gender			
Female	119 (32.6%)	50 (24.6%)	169 (29.8%)
Male	246 (67.4%)	153 (75.4%)	399 (70.2%)
TNM stage			
I	170 (46.6%)	33 (16.3%)	203 (35.7%)
II	84 (23"0%)	96 (47.3%)	180 (31"7%)
III	83 (22.7%)	59 (29.1%)	142 (25.0%)
IV	4 (1.1%)	15 (7.4%)	19(3.3%)
Unknow	24 (6.6%)	0 (0%)	24 (4.2%)
Survival status			
Alive	234(64.1%)	168 (82.8%)	402 (70.8%)
Dead	130 (35.6%)	35 (17.2%)	165 (29.0%)
Not reported	1 (0.3%)	0 (0%)	1 (0.2%)
OS time (days)			
Mean (SD)	813 (726)	828(417)	818 (633)
Median	596	810	661

*HCC* hepatocellular carcinoma, *TCGA* the cancer genome atlas, *ICGC* international cancer genome consortium

### Clustering analysis for disulfidptosis-related genes

Sixteen disulfidptosis-related genes [including 4 confirmed suppressors of disulfidptosis (NCKAP1, RPN1, SLC3A2 and SLC7A11) and 12 potential synergistic of disulfidptosis (PPM1F, CNOT1, NDUFC1, NDUFA11, NDUFA10, LRPPRC, NUBPL, NDUFS2, GYS1, EPAS1, NDUFS1 and OXSM)] were obtained from the previous studies of Liu et al. in 2023 [5]. These DRGs can be seen in Additional file 2: Table S1. We conducted the consensus clustering analysis using the ConsensusClusterPlus R package, (parameters of reps = 1000), to assess the disulfidptosis status and DRG cluster clusters of HCC patients in the TCGA-LIHC cohort. We select a suitable parameter for clusters through the CDF curve. Then, the patient OS time was determined through Kaplan–Meier (K-M) analysis using the “survival” and “survminer” packages.

### Identification of DEGs and GSEA

Differential gene expression (DEGs) analysis between two disulfidptosis regulated clusters was performed using an empirical Bayesian algorithm using the “limma” R package and DEGs were given a significance threshold of |log2 (FoldChange)|> 1 and an adjusted P-value of 0.05. Applying the ‘‘clusterProfiler’’ packages, gene set enrichment analysis (GSEA) and gene ontology (GO) enrichment analyses were carried out to study the biological activities of two disulfidptosis regulated clusters [11]. Annotation files for GO, the Kyoto Encyclopedia of Genes and Genomes (KEGG), and hallmark pathways were downloaded from the GSEA website (http://www.gsea-msigdb.org/gsea/index.jsp).

### Construction and validation of prediction models

The univariate Cox proportional hazards regression survival analysis was performed via the ‘‘survival’’ R package to filter prognostic genes as in the TCGA-LIHC cohort based on the DEGs between two disulfidptosis regulated clusters. The prognostic genes were then further filtered to identify hub DRGs genes using the least absolute shrinkage and selection operation (LASSO) and tenfold cross validation using the ‘‘glmnet’’ R package. The sum of the expression value and weight coefficients of the hub genes was then used to calculate the DRG scores for each sample:

$$\mathrm{DRG scores}={\sum }_{k=1}^{n}\left(\genfrac{}{}{0pt}{}{n}{k}\right){LASSO\_coef}^{k}{*Expression}^{k}$$

To evaluate the survival probabilities between the two DRG score groups in the TCGA training cohort, a Kaplan‒Meier (K‒M) analysis was conducted using the ‘‘survival’’ and ‘‘survminer’’ packages. The area under the curve (AUC) was calculated using the ‘‘timeROC’’ package, which also performed receiver operating characteristic (ROC) analysis across 1, 3, and 5 years. Similarly, K‒M survival curves and ROC curves were generated for the ICGC-LIRI-JP testing cohort. Then, using clinical traits and DRG scores, multivariate Cox proportional-hazards regression was used to identify the independent predictors of HCC prognosis. Finally, using the ‘‘rms’’ R package, the stage and DRG scores served as independent predictors to generate a nomogram for clinical application. [12]. To assess the nomogram model, the ‘‘ggDCA’’ R package implemented the decision curve analysis (DCA).

### Immune feature evaluation of DRG scores

Each HCC patient in the TCGA-LIHC cohort was assigned an immune score, stromal score, and an ESTIMATE score using the ESTIMATE method. The infiltration score of 23 human immune cells in each HCC patient was calculated via the CIBERSORT algorithm [13]. Additionally, Spearman correlation analysis was used to determine whether immune checkpoint genes and DRG scores in HCC patients were correlated.

### Analysis of drug susceptibility and immunotherapy response

For HCC patients in the TCGA-LIHC cohort, the Tumor Immune Dysfunction and Exclusion (TIDE) algorithm was used to forecast the immunotherapy response (http://tide.dfci.harvard.edu) [13]. The ‘‘oncoPredict’’ package was used to calculate the half-inhibitory concentration (IC50) values for 198 medicines, including cancer treatment and medications specifically for HCC. [14].

### scRNA-seq data analysis

Single-cell data of seven tumors of untreated HBV-related hepatocellular carcinoma (HCC) patients were downloaded from the GEO website (GSE202642) [15]. The ‘‘Seurat’’ R package was used for standardizing the single-cell analysis workflow. Cells expressing a minimum of 200 genes with mitochondrial percentage less than 10% were selected, followed by normalization and scaling. Subsequently, the RunPCA function was employed to perform principal component analysis (PCA), and the top 15 PCs were chosen for uniform manifold approximation and projection (UMAP) reduction. Cell type annotations were performed based on the marker genes of the major cell clusters. Finally, the AddModuleScore function was used to generate the DRG signature at the single-cell level.

### Cell culture

The Cancer Research Institute of Central South University provided HepG2, L-02, SMMC-7721, HCCLM3 and Hep3B cells, as well as 293 T cells. All cell lines were cultured at 37 °C in a 37 ℃, 5% CO_2_ incubator in DMEM medium (Gibco) with 10%(v/v) BCS (Sigma) and 1%(v/v) streptomycin/penicillin/gentamicin. Furthermore, the above cell lines were free of mycoplasma contamination and were passaged < 10 times after being revived from frozen stock.

### Plasmid constructs

The overexpression (OE) plasmid pEnter-LCAT was purchased from Vigene Biosciences (CH896503). Moreover, all constructions were confirmed by DNA sequencing. Subsequently, cells were transfected with the LCAT or vector plasmids using LipoMax DNA Transfection Reagent (SUDGEN, 32,110) according to the manufacturer's procedure to establish transduced cell lines. After transfection for 6 h, the medium was replaced with medium supplemented with 10% (v/v) BCS. After that, we verified the expression of LCAT in the cells via Western blotting.

### Quantitative real-time PCR assays

As previously mentioned [16, 17], quantitative real-time PCR experiments were carried out. In brief, the PrimeScriptTM RT Reagent Kit with gDNA Eraser (TaKaRa, Kusatsu, Japan) was used for reverse transcription after total RNA was extracted using RNAiso Plus (TaKaRa, Kusatsu, Japan) reagent. Afterward, real-time PCR was performed using a Bio-Rad CFX Connect real-time PCR apparatus. By deducting the Ct values of beta-actin (ACTB), the Ct values of each gene were normalized. Additional file 2: Table S2 contains a list of the primers.

### Western blot analysis

The collected cells were washed twice with PBS buffer washed before being lysed with IP lysis buffer containing protease inhibitor cocktail for two hours on ice. The systems were set up after 15 min of centrifugation at 15,000 rpm to determine the supernatant (BCA protein assay). Total protein was isolated using SDS–polyacrylamide gel and then transferred to a polyvinylidene fluoride (PVDF) membrane. The membranes were incubated overnight with different primary antibodies at 4 °C, incubated with the corresponding secondary antibody for 2 h at room temperature, and exposed via the ChemiDox XRS + image-forming system. The following antibodies were used: anti-LCAT (Baijia; IPB11128), and anti-β-actin (Sigma; cat #A5411).

### Cell viability, colony formation and transwell assays

Cell proliferation, colony formation, and Transwell experiments were conducted, as previously reported [18]. In brief, the cell Counting Kit-8 (CCK-8) test was used for the cell viability experiment in accordance with the manufacturer’s instructions (Bimake). First, 1000 cells were plated in 96-well plates with five parallel wells in each group. Next, 10 μL of CCK-8 reagent was added to each well at each time point (0 h, 24 h,48 h,72 h,96 h), and each well was then incubated for 2 h at 37 ℃ with 5% CO_2_ for 2 h, and the OD450 was detected (BioTek).

In addition, cells were seeded onto six-well plates at a density of 500 cells per well for the colony formation experiment, and the plates were then incubated for 2 weeks. Each cell was seeded with three parallel wells. Then, the cells were fixed with methanol for 10 min, stained with 0.5% crystal violet for 30 min and subsequently counted using a microscope and ImageJ software (1.47 v, NIH, USA).

In the Transwell migration assay, 5 × 10^4^ cells were plated in each 24 well chamber (8.0 μm pore size, Falcon) and incubated for 24 h. For the invasion test, 100 μl of Matrigel (Becton, Dickinson and Company, USA) was diluted 1:9 with serum-free media and placed onto transwell chambers. Each compartment received 1 × 10^5^ cells, which were then cultivated for 48 h later. After that, the cells were fixed with methanol, stained with 0.5% crystal violet for 30 min and examined under an optical microscope. Each of these assays was repeated at least three times.

### Immunofluorescence analysis

Cells were placed on chamber slides and then were washed three times with PBS. Subsequently, the were permeabilized with 0.5% Triton X-100 (v/v) for 5 min at room temperature after fixation with 4% paraformaldehyde for ten min. For each slide, 100 nM Actin-stain™ 555 (Cytoskeleton, PHDH1) in PBS was added and incubated at RT for half an hour in the dark. Next, the slides were contained with DAPI for 10 min, and washed 3 times with PBS. Finally, a Zeiss LSM9 confocal microscope was used to capture fluorescence images.

## Results

### Development of disulfidptosis-regulated clusters and their features in HCC

**Fig. 2:**
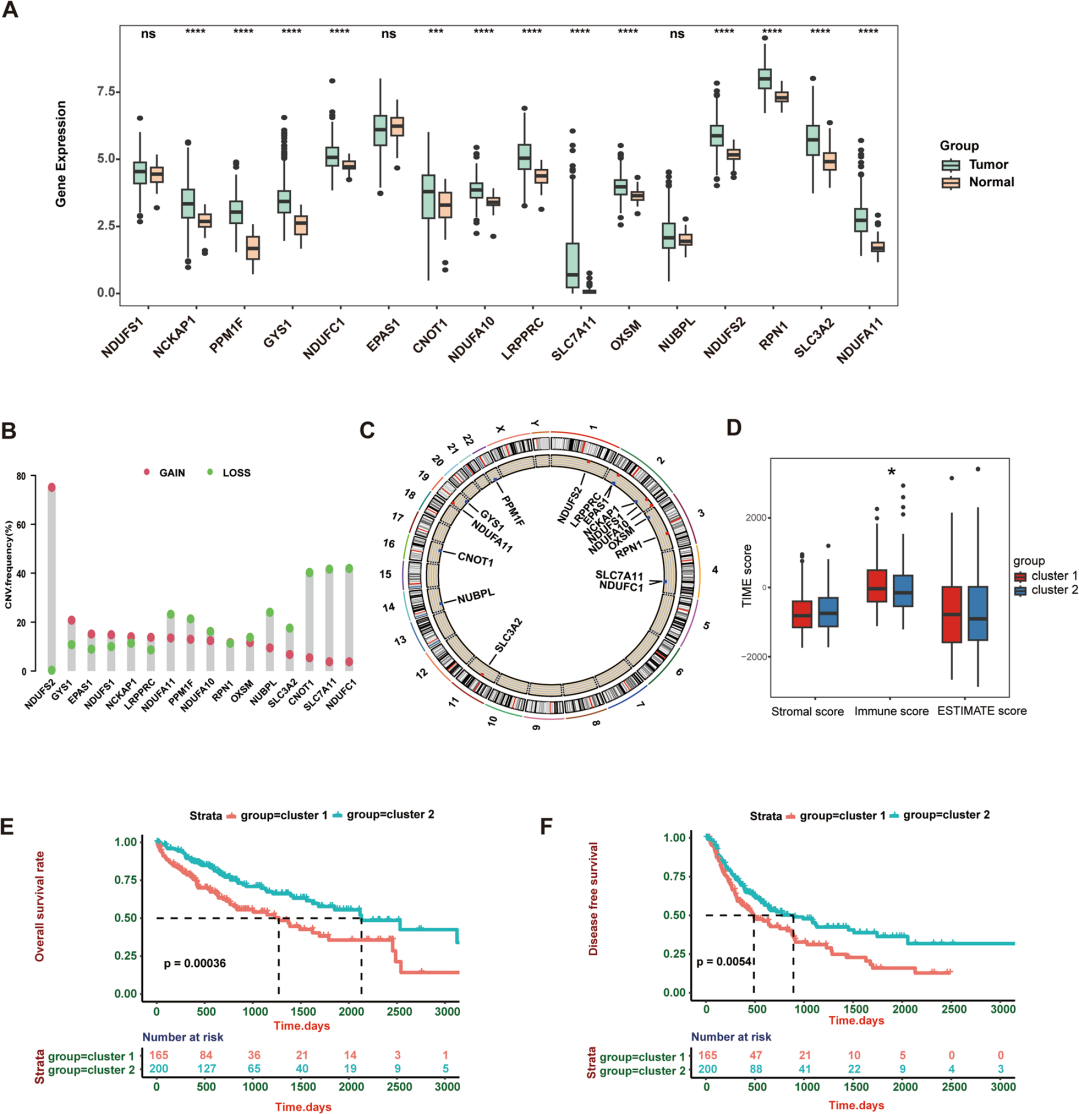
Identification of disulfidptosis-regulated clusters based on 16 DRGs in HCC. **A** Differences in the expression levels of 16 DRGs in HCC and normal tissue, most of those genes were significantly upregulated in HCC tissues. **(B)** CNV (amplification and deletion) frequencies of DRGs in HCC. **(C)** The location of CNVs of DRGs on 23 chromosomes. **(D)** Boxplot showing the significant difference in the immune score between the two disulfidptosis-regulated clusters in the TCGA cohort (Wilcoxon test, * *P* < 0.05). **(E **and** F)** The prognosis analysis for two disulfidptosis-regulated clusters: Cluster 1 had lower overall survival (OS) and progression-free interval (PFI) than Cluster 2. *P* values are shown as * *P* < 0.05; ** *P* < 0.01; *** *P* < 0.001; ns, not significant. *DRGs* Disulfidptosis-related genes, *HCC* hepatocellular carcinoma, *CNV* copy number variation, *OS* overall survival, *PFI* progression-free interval, *TIME* tumor immune microenvironment

We proceeded by analyzing the expression of DRG in the tumor tissues and adjacent tissues of TCGA cohort HCC cases. Except for NDUFS1, EPAS1 and NUBPL, the expression of the other 13 DRGs including NCKAP1, PPM1F, GYS1, NDUFC1, CNOT1, NDUFA10, LRPPRC, SLC7A11, OXSM, NUDFS2, RPN1, SLC3A2 and NDUFA11, was significantly upregulated in HCC tissues (Fig. 2A). The frequency of CNVs in 16 DRGs from HCC was next examined; most DRGs showed copy number amplification and deletion (Fig. 2B). The locations of the CNV changes for 16 DRGs on 23 chromosomes are shown in Fig. 2C. We classified HCC patients in the TCGA dataset using a consensus clustering approach in order to further define the expression signatures of DRG in HCC patients. The clustering results showed that when k = 2 was optimal (Additional file 1: Figure S1A–F), and these patients were well divided into two clusters (165 in Cluster 1, 200 patients in Cluster 2). Using ESTIMATE algorithms to evaluate the immune infiltration of the two clusters, we found that compared to cluster 2, cluster 1 had a higher immune score, suggesting the potential immune regulation function of DRGs in HCC (Fig. 2D). Then, the Kaplan–Meier analysis showed that patients in Cluster 1 had a lower overall survival (OS) rate and a shorter progression-free interval (PFI) than those in Cluster 2 (Fig. 2E, F).

### Construction of the disulfidptosis-related prognostic model

**Fig. 3:**
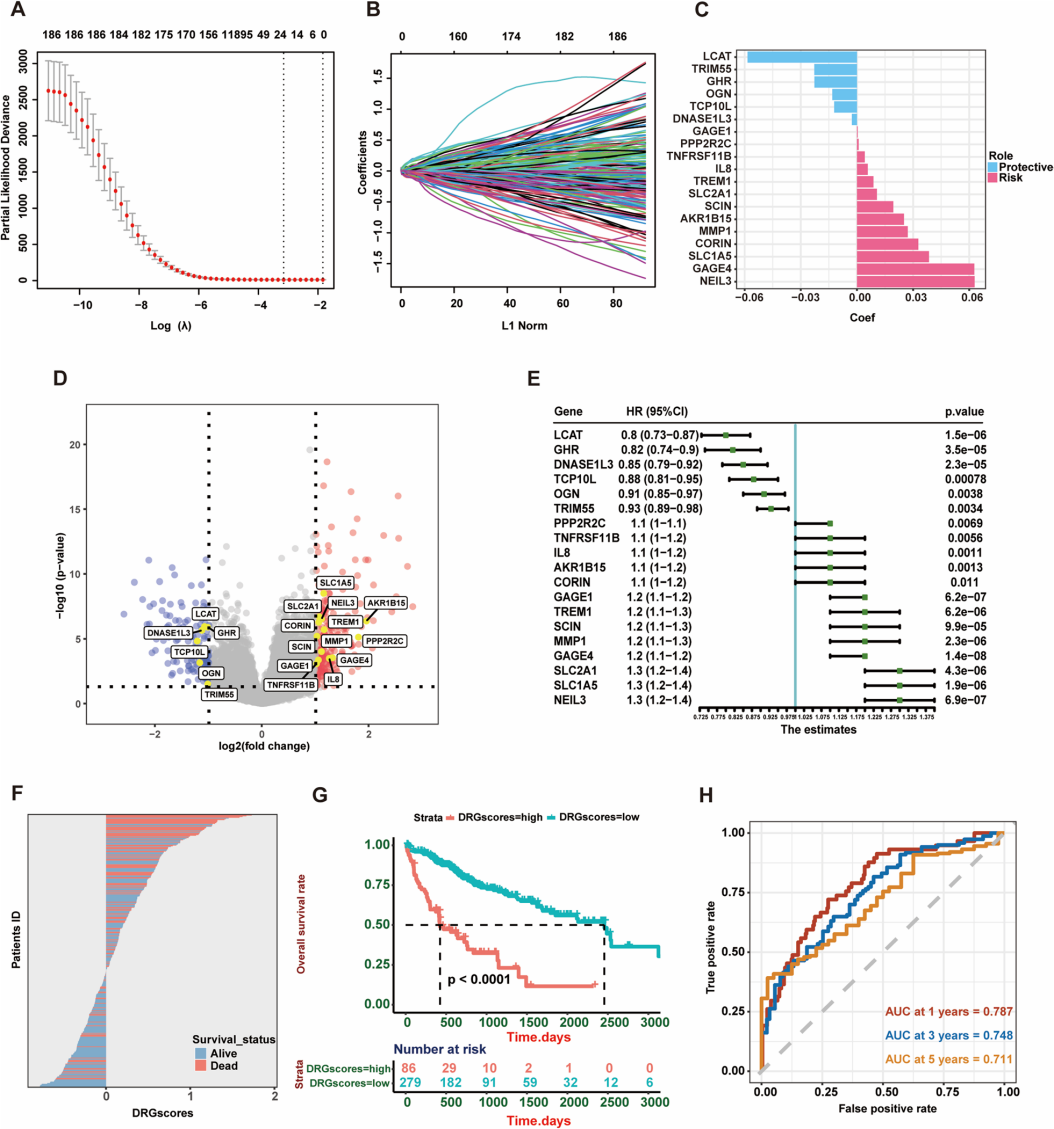
Construction of the disulfidptosis-related prognostic model in HCC. **A** and B LASSO regression identified hub DRGs and coefficient profiles. **C** Histogram showing the 6 protective genes and 13 risk genes and their weighted coefficients. **D** Volcano plot of 19 selected hub genes between two disulfidptosis-regulated clusters. **E** Univariate Cox regression analysis of selected hub genes associated with overall survival in HCC patients. **F** Distribution of DRG scores and patient survival status in the TCGA-LIHC cohort. **G** and **H** The Kaplan–Meier OS curves **G** and time-dependent ROC curves **H** for patients in the high- and low-DRG score groups in the TCGA training cohort. The group with high DRG scores had worse survival rates, and the AUC values of DRG scores for one-year, three-year, and 5 year OS were 0.787, 0.748, and 0.711, respectively. *DRGs* Disulfidptosis-related genes; *HCC* hepatocellular carcinoma, *LASSO* least absolute shrinkage and selection operator, *DEGs* differentially expressed genes, *LIHC* liver cancer, *ROC* receiver operating characteristic

We used the ‘‘limma’’ R package to analyze the differences in gene expression between the two clusters. A total of 373 DEGs were screened out, and after further using univariate Cox regression analysis to select those genes with prognostic significance, a total of 190 DEGs were included in the following analysis (Additional file 2: Tables S3, S4). The majority of metabolism-related pathways, including those for amino acid metabolism and fatty acid metabolism, were repressed in Cluster 1 according to GSEA of hallmark pathways, KEGG pathway analysis, and GO enrichment analysis based on the DEGs (Additional file 1: Figure S2A–C). On the other hand, immune-related pathways, such as the inflammatory response and TNF-α signaling via the NF-κB pathway, were activated in Cluster 1, suggesting differences in immune status and metabolism regulation between these two disulfidptosis clusters. The best λ value was then determined using LASSO regression analysis, and 19 hub genes were determined (Fig. 3A, B). Among these hub genes, LCAT, TRIM55, GHR, OGN, TCP10L and DNASE1L3 were identified as protective genes, while GAGE1, PPP2R2C, TNFRSF11B, IL8, TREM1, SLC2A1, SCIN, AKR1B15, MMP1, CORIN, SLC1A5, GAGE4 and NEIL3 were identified as risk-related genes (Fig. 3C). The volcano plot showed that all the risk genes were significantly upregulated and the protective genes were significantly downregulated in Cluster 1 (Fig. 3D). The results of the univariate analysis indicated the adverse or protective effects of these hub genes on patient survival in Cluster 1 (Fig. 3E). Then, we constructed the disulfidptosis-related gene score model based on the LASSO regression results: DRG score = (− 0.05847)* LCAT + (-0.02276)* TRIM55 + (-0.02275)* GHR + (− 0.01317)* OGN + (− 0.01217)* TCP10L + (-0.0027)* DNASE1L3 + 0.00039* GAGE1 + 0.0009* PPP2R2C + 0.00413* TNFRSF11B + 0.00576* IL8 + 0.00878* TREM1 + 0.01065* SLC2A1 + 0.01942* SCIN + 0.02512* AKR1B15 + 0.02715* MMP1 + 0.03276* CORIN + 0.0385* SLC1A5 + 0.0627* GAGE4 + 0.06288* NEIL3. In the TCGA-LIHC training cohort, patients with high a DRG score had a substantially worse survival rate (Fig. 3F, G). In the training cohort, the DRG score had satisfactory predictive value (AUC for survival at 1 year, 3 years, and 5 years were 0.787, 0.748, and 0.711, respectively; Fig. 3H).

### Validation of the prognostic model and establishment of a disulfidptosis-related predictive nomogram for HCC

**Fig. 4:**
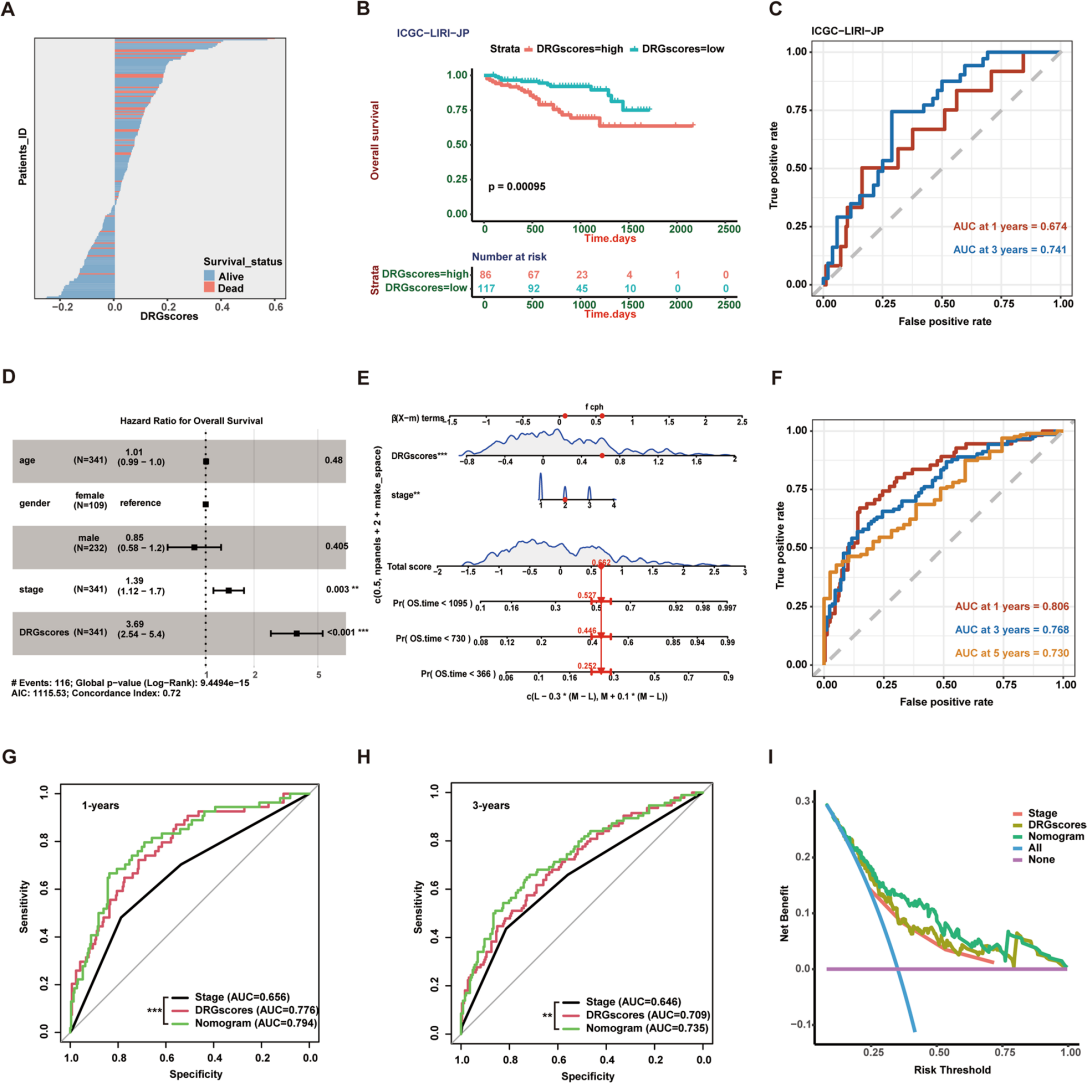
Validation of the DRG score model and establishment of a disulfidptosis predictive nomogram for HCC. **A–C** Distribution of patient survival status **A** and Kaplan–Meier OS curves **B** and time-dependent ROC curves **C** for patients in the high- and low-DRG score groups in the ICGC − LIRI-JP validation cohort. The group with high DRG scores had a worse survival rates (*P* = 0.00095), and the AUC values of the DRG scores for 1 year and 3 year OS were 0.674 and 0.741, respectively. **D** Multivariate Cox regression showed that high stage (*P* = 0.003, HR = 1.39) and DRG score (*P* < 0.001, HR = 3.69) were independent risk factors for worse OS. **E** The establishment of a nomogram to predict the 1-, 3-, and 5 year overall survival probability for HCC patients in the TCGA cohort. **F** The ROC curves demonstrated that the nomogram model's AUC values for one-year OS, 3 year OS, and 5 year OS were 0.806, 0.768, and 0.730, respectively. **G** and **H** Compared with stage or DRG score alone, the nomogram model showed a higher AUC value for 1 year **G** and three-year **H** OS, which suggested that the model was effective. **I** The DCA curve suggested that the nomogram model performs better in clinical practice than stage or DRG score alone. *P* values are shown as * *P* < 0.05; ** *P* < 0.01; *** *P* < 0.001; *ns* not significant. *DRGs* Disulfidptosis-related genes, *ROC *receiver operating characteristic, *ICGC* international cancer genome consortium, *HCC* hepatocellular carcinoma, *AUC* area under curve, *DCA* decision curve analysis

Next, we chose the ICGC-LIRI-JP liver cancer cohort as our test cohort to validate our prognostic model. Similar to our previous analysis, with the increase in DRG score, the risk of patient death increased, and the survival time decreased. K‒M survival curves revealed that the high DRG score group had poorer overall survival (Fig. 4A, B). Importantly, the one-year and three-year survival predictions based on the DRG score model had AUC values of 0.674 and 0.741, respectively, in the ICGC-LIRI-JP validation cohort (Fig. 4C). Then, we performed multivariate Cox regression analyses on the clinical characteristics of the TCGA-LIHC cohort and the DRG score and found that DRG score and stage can be used as independent prognostic predictors for HCC patients (Fig. 4D). We further integrated the DRG score and stage to create a nomogram in the TCGA-LIHC cohort to create individualized predictions for HCC patients (Fig. 4E). Furthermore, the nomogram model's AUC value for patient OS accuracy was better (AUC for 1 year, 3 years, and 5 years of survival were 0.806, 0.758, and 0.730, respectively; Fig. 4F). Importantly, compared with stage or DRG score alone, the nomogram model showed higher predictive value for patient survival status (Fig. 4G, H). The DCA curve also suggested that the nomogram model performs better in clinical practice than stage or DRG score alone (Fig. 4I).

### Evaluation of tumor microenvironment features based on the DRG scores

**Fig. 5:**
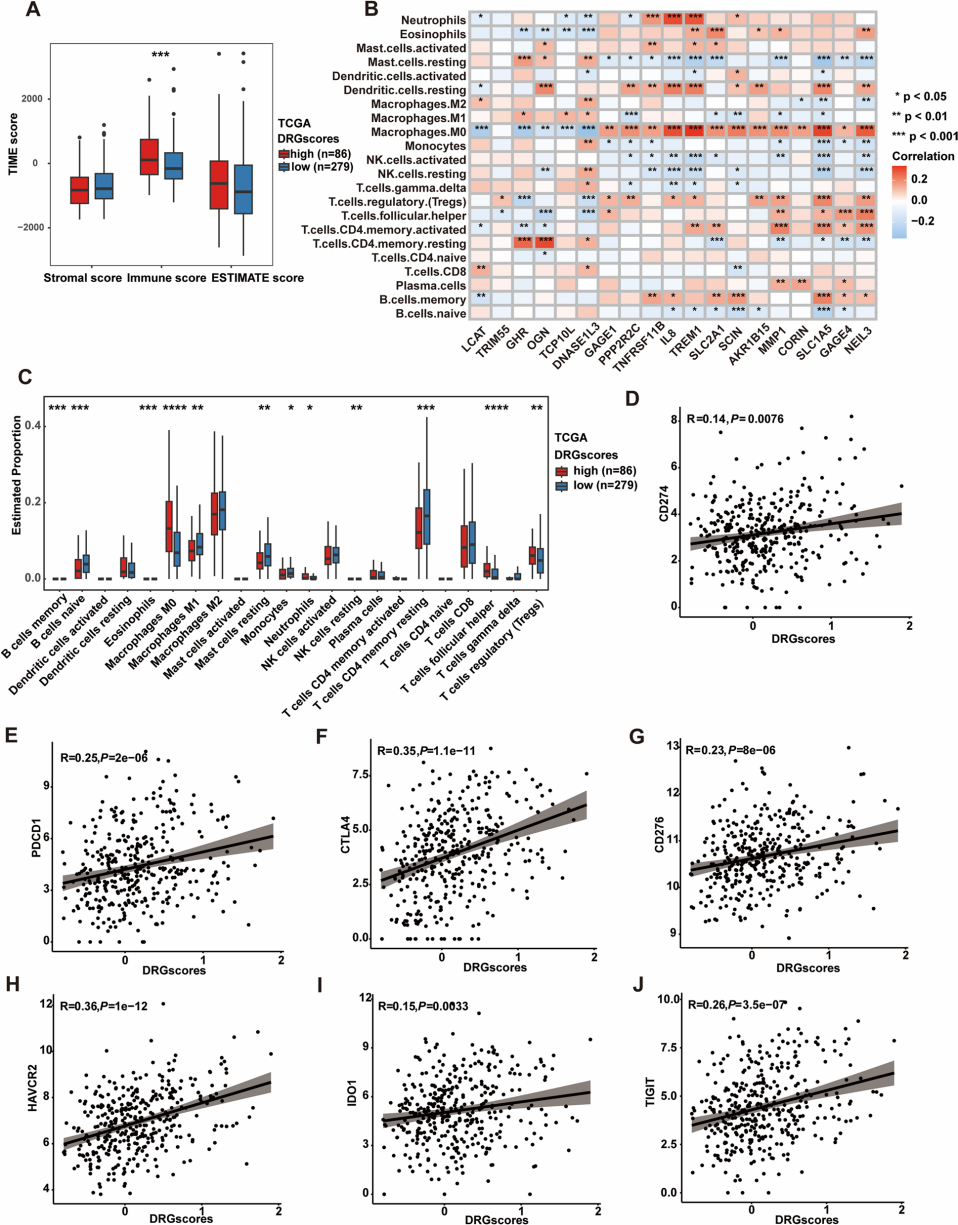
Evaluation of tumor microenvironment (TME) features based on the DRG score. **A** Boxplot showing that the immune score of the high-DRG group was significantly higher than that of the low-DRG score group in the TCGA cohort (Wilcoxon test, *** *P* < 0.001). **B** CIBERSORT analysis of correlations between the proportion of immune cells and 19 hub genes showed that these genes in HCC patients in the TCGA cohort were closely related to macrophages, B cells, NK cells and T cells. **C** Boxplot showing the proportion of immune cells in the low- and high-DRG score groups in HCC patients, which suggested that the high DRG score group had higher infiltration levels of macrophages and Tregs and lower infiltration levels of naive B cells, CD4 + T cells and CD8 + T cells. **D–J** The immune checkpoint genes (CD274, PDCD1, CTLA4, CD276, HAVCR2, TIGIT and IDO1) significantly correlated with the DRG score. *P* values are shown as * *P* < 0.05; ** *P* < 0.01; *** *P* < 0.001; ns, not significant. *HCC* hepatocellular carcinoma, *TME* tumor microenvironment, *DRGs* Disulfidptosis-related genes

We initially employed ESTIMATE methods to compare the immune infiltration features of the two patient groups with high and low DRG scores in order to better understand the characteristics of the immunological milieu of the two patient groups. Patients with high and low DRG scores had significantly different immune scores (Fig. 5A). Using the CIBERSORT method, we further examined the relationship between 19 hub genes and different immune cells and found that the expression of these genes in the TCGA-LIHC cohort was closely related to the infiltration levels of macrophages, NK cells and T cells (Fig. 5B). Moreover, we also found that in the high DRG score group, the infiltration levels of macrophages and Tregs were higher, while the infiltration levels of naive B cells, CD4 + T cells and CD8 + T cells were lower (Fig. 5C), suggesting that the high DRG score group had a suppressive immune microenvironment, which coincided with their poorer overall survival outcomes. In addition, we analyzed the correlation between the expression of immune checkpoint genes (CD274, PDCD1, CTLA4, CD276, HAVCR2, TIGIT and IDO1) and DRG score in the TCGA-LIHC cohort [19, 20]. We observed a significant positive correlation between the immune checkpoint genes and DRG score (Fig. 5D–J). Analysis of the features of hub genes at the single-cell level to better understand the effectiveness of the constructed model has become a commonly employed method in recent years [21]. We analyzed the single-cell data of untreated hepatitis B virus (HBV)-related hepatocellular carcinoma (HCC) patients (GSE202642) [15]. Following the study conducted by Zhu et al., 9 cell clusters were identified (Additional file 1: Figure S3A, B). Subsequently, we investigated the expression of our hub DRGs within each cell cluster. The results indicated that the DRG signature was predominantly distributed on tumor cells, endothelial cells, cancer-associated fibroblasts (CAFs), tumor-associated macrophages (TAMs), and T cells, which corresponded to our CIBERSORT immune infiltration analysis results.

### Correlations of the DRG scores with immunotherapy and drug sensitivity

**Fig. 6:**
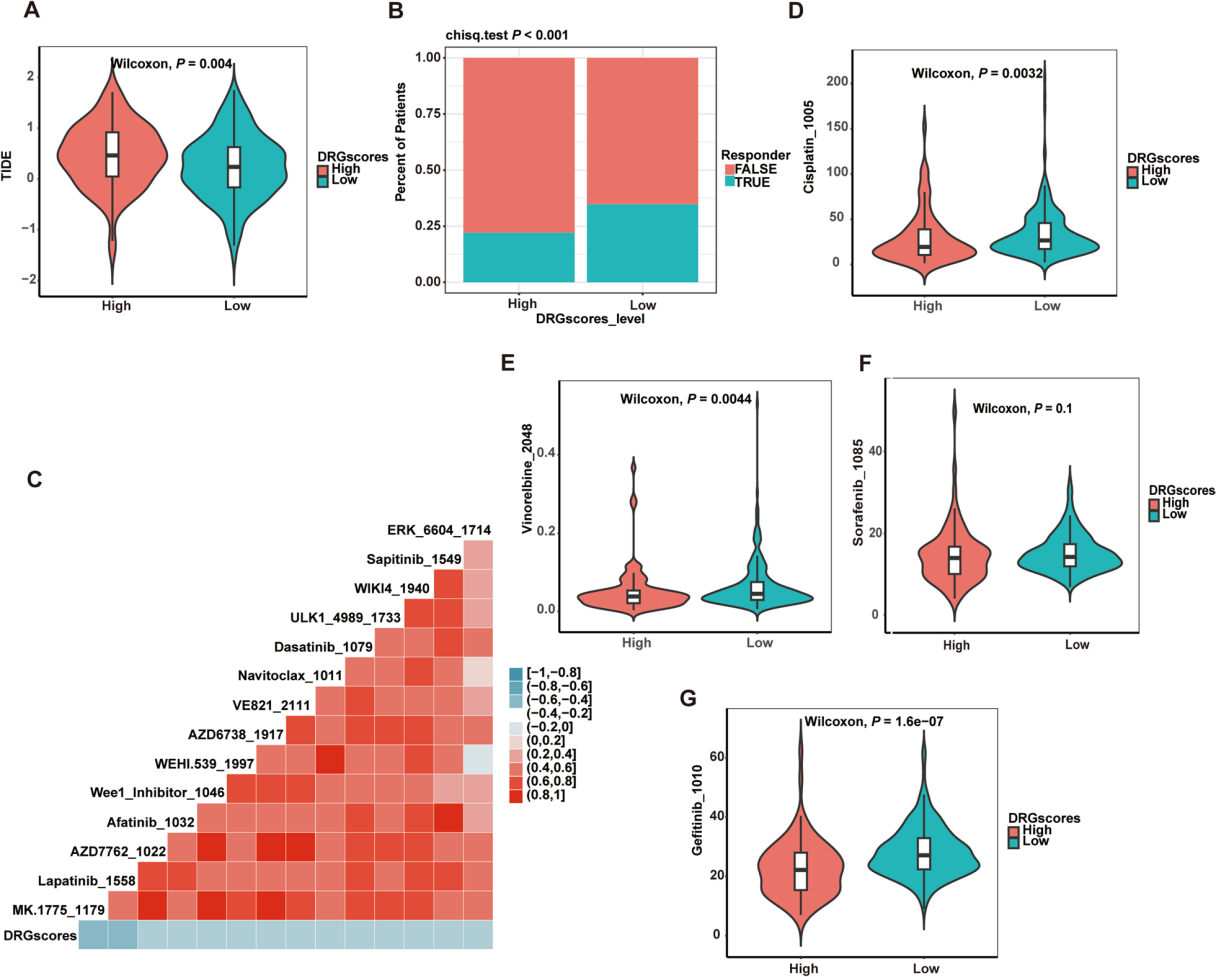
Correlations of the DRG score with immunotherapy and chemotherapeutic sensitivity in HCC. **A** Compared to patients in TCGA-LIHC with low DRG scores, those in the high DRG score group exhibited higher TIDE scores (Wilcoxon, P < 0.01). **B** The response rate of the high DRG score group was significantly lower than that of the low DRG score group (chi sq. test P < 0.001). **C** Correlations of the DRG scores and drug sensitivity in patients (Spearman correlation < − 0.2). The therapeutic sensitivity of cisplatin **D**, vinorelbine **E**, sorafenib **F**, and gefitinib **G** between the high- and low-risk groups. *DRGs* Disulfidptosis-related genes, *TIDE* Tumor immune dysfunction and exclusion

We then applied the TIDE algorithm to evaluate the immunotherapy response for HCC patients in the TCGA-LIHC cohort. According to our results (Fig. 6A, B), patients with low DRG scores may benefit more from immunotherapy because their TIDE scores were significantly higher than those of the low DRG score group and their response rates were significantly lower than those of the high DRG score group. We further predicted the efficacy of 198 drugs by the oncoPredict R package in TCGA-LIHC and assessed the drug sensitivity of the high- and low-DRG score groups. Forty drugs were negatively correlated with DRG scores (*P* < − 0.2, Additional file 2: Table S5), and Fig. 6C shows the top ten drugs with a negative correlation. Chemotherapy drugs for liver cancer, such as cisplatin and vinorelbine, and the targeted drugs gefitinib and sorafenib had lower IC50 values in patients with high DRG scores (Fig. 6D–G), suggesting that the above drugs have better therapeutic effects on these patients. Overall, the findings showed that the prognostic model based on DRG scores can aid in predicting the efficacy of medications in HCC patients.

### Validation of the DRG hub genes in HCC cell lines and functional analysis

**Fig. 7:**
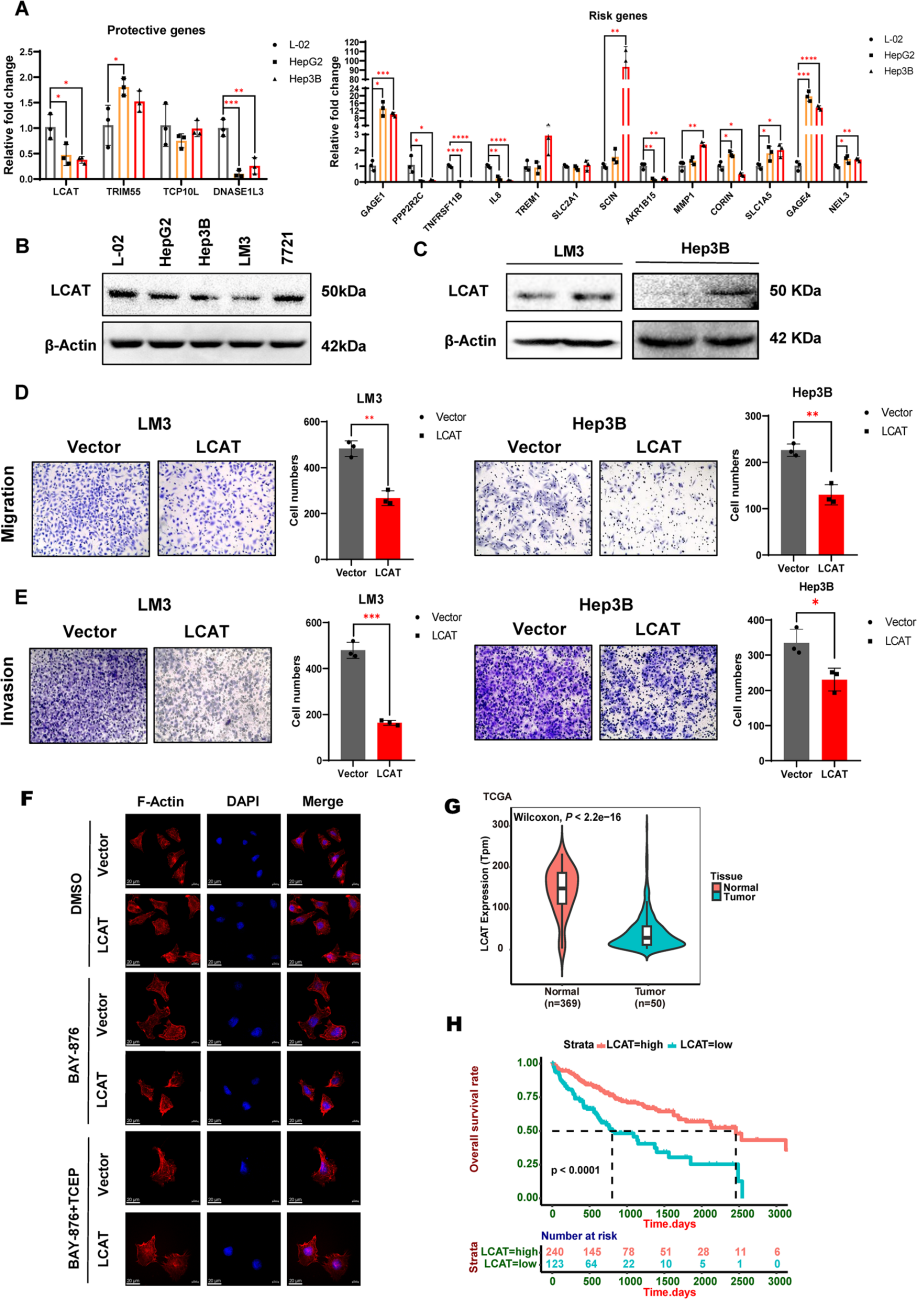
Validation of the hub DRGs in HCC cell lines and functional analysis. **A** qPCR was used to analyze the mRNA expression of the protective and risk-related genes for disulfidptosis model in liver cancer cells. **B** Western blotting analyses of LCAT expression in liver cancer cell lines. **C** Western blotting analyses of LCAT expression in LM3 and Hep3B cell lines. **D** Migration was measured in LCAT-overexpressing LM3 and Hep3B cells. **E** Invasion was measured in LCAT-overexpressing LM3 and Hep3B cells. **F** Fluorescence staining of F-actin with phalloidin in vector and *LCAT*-OE LM3 cells treated with 5 μM BAY-876 or 1 mM TCEP for 18 h. **G** and **H**: The expression of LCAT in tumor tissues was significantly lower than that in adjacent nontumor tissues in HCC patients in the TCGA cohort **G**, and patients with higher LCAT expression demonstrated better survival outcomes **H**

We initially used qPCR to assess the mRNA expression levels of hub DRGs in two liver cancer cell lines (HepG2 and Hep3B) and a normal liver cell line (L-02) to further validate our investigation. The results demonstrated that LCAT and DNASE1L3 were significantly downregulated in liver cancer cells, while NEIL3, GAGE4, SLC1A5, MMP1, SCIN and GAGE1 mRNA expression levels were notably elevated (Fig. 7A), in agreement with the bioinformatics prognostic model described above. The mRNA expression levels of a few hub genes were too low in the liver cancer cell line to be detected by qPCR. Since the protective gene LCAT has the lowest risk coefficient for hepatocellular carcinoma, we further explored the expression profile and identified the biological function of LCAT. Firstly, Western blotting analysis revealed that liver cancer cells expressed less of the LCAT protein than normal liver cells did (Fig. 7B, Additional file 1: Fig. S4A). After that, to examine the impact of LCAT overexpression in HCC, we established LCAT overexpressing HCCLM3 and Hep3B cell lines (Fig. 7C, Additional file 1: Figure S4B, C). Transwell assays indicated that increasing LCAT expression dramatically reduced the capacity of LM3 and Hep3B cells to migrate and invade (Fig. 7D, E). However, LCAT did little to suppressed the cell proliferation (Additional file 1: Fig. S4D, E) and colony formation (Additional file 1: Fig. S4F, G) capabilities of liver cancer cells in vitro. Thus, we inferred that LCAT is primarily associated with liver cancer metastasis. Moreover, we treated LM3 cells with BAY-876 (Selleck, S8452), a GLUT1 inhibitor agent, to block glucose uptake. Immunofluorescence results showed that overexpressing LCAT in LM3 cells during glucose deprivation obviously altered their cell shape, including F-actin contraction and cell shrinkage (Fig. 7F). Furthermore, the reducing drug TCEP (Selleck, S4611) was successful in preventing disulfidptosis-induced cell death. Hence, LCAT enhanced glucose starvation-induced disulfidptosis of liver cancer cells through actin cytoskeletal network breakdown. Finally, we analyzed the expression differences of LCAT in HCC patient tumor tissues and adjacent nontumor tissues in TCGA. Then, we generated the survival curves for patients with high LCAT expression (n = 240) and low LCAT expression (n = 123). The results revealed a significantly lower expression of LCAT in cancer tissues than in adjacent nontumor tissues (Fig. 7G). Furthermore, patients with high LCAT expression exhibited a better survival status (Fig. 7H). These findings also suggest that LCAT may serve as a protective gene, suppressing the progression of liver cancer. Our findings collectively suggest that LCAT slows the progression of HCC by mediating disulfidptosis.

## Discussion

Disulfidptosis, as a newly discovered form of PCD, is closely associated with cancer metabolism and provides new insights for cancer treatment. However, the specific role of this cell death pathway in various types of tumors remains poorly understood, and its relationship with hepatocellular carcinoma (HCC) is still unclear.

In our research, we selected 16 genes critical for mediating and inhibiting the occurrence of disulfidptosis as disulfidptosis-related genes. Considering that disulfidptosis is an induced process in cancer cells, analysis of genes involved in this process could accurately and fundamentally reflect the disulfidptosis status of each patient, and our data support the reliability of the model. From the DRGs, we identified 19 hub genes to construct our model. Among these genes, 6 genes were identified as protective genes and the remaining 13 genes were identified as risk-related genes. Importantly, we validated the expression of some genes in liver cancer and normal liver tissue cell lines through RT‒qPCR experiments.

We further selected lecithin-cholesterol acyltransferase (LCAT) for biological functional experiments. LCAT is involved in cholesterol transport and converts free cholesterol into its hydrophobic form, ultimately synthesizing high-density lipoprotein (HDL) [22]. LCAT is abundantly expressed in the human body and has low expression in breast cancer and liver cancer tissue that in compared to normal tissue, but high expression in the serum of advanced breast cancer, suggesting that LCAT may serve as a plasma protein biomarker for late-stage breast cancer and metastasis [23]. In contrast, LCAT levels are lower in the serum of colorectal cancer, liver cancer, and ovarian cancer patients, making it a potential biomarker [24–26].

In addition, bioinformatics analysis has indicated that LCAT plays a critical role in the development of liver cirrhosis into HCC [27]. Our experiments have confirmed the low expression of LCAT in liver cancer cell lines, consistent with previous reports [28]. Furthermore, in vitro experiments have clearly demonstrated that LCAT significantly inhibits the migration and invasion abilities of liver cancer cells, and promotes cell death via disulfide bond formation under glucose deprivation conditions. However, LCAT has a limited impact on the proliferation and colony formation abilities of liver cancer cells, suggesting that LCAT mainly affects the metastasis of liver cancer by inducing disulfidptosis. Then, the role of LCAT in disulfidptosis need further experimental verification. Since LCAT is a newly discovered tumor suppressor, it is worthwhile to further investigate the molecular mechanisms by which LCAT affects disulfidptosis in HCC.

Moreover, our results demonstrated that the developed DRG score model successfully divides HCC patients into two clusters. The group with high DRG scores had a worse prognosis and exhibited an immunosuppressive microenvironment characterized by immune infiltration. Additionally, this group showed stronger resistance to immune therapy. However, these patients demonstrated greater sensitivity to certain chemotherapy drugs for liver cancer, such as cisplatin and vinorelbine. Immune cells, particularly CD4 + T cells, CD8 + T cells, and Th cells, play critical roles in a variety of diseases, including cancer and several infectious diseases [29–31], and some costimulatory molecule and immune check point such as CD40 and PD-1 are extensively expressed abnormally in different immune cells [32, 33]. In our study, CIBERSORT analysis showed that DRG hub genes are closely related to T cells. Patients with high DRG scores have lower CD4 + T cell and CD8 + T infiltration, and DRG scores are positively correlated with the expression of immune checkpoint genes such as PD-1. These results also suggested that patients with high DRG scores may be more sensitive to immunotherapy.

Currently, sorafenib, which targets ferroptosis in liver cancer cells, is the most useful first-line chemotherapy drug for the treatment of advanced HCC [34, 35]. Moreover, regulating ferroptosis could also alleviate the tumor immunosuppressive microenvironment, thereby enhancing the sensitivity of patients to immunotherapy [36]. In addition, applying AEG35156, resminostat (4SC-201) or panobinostat (LBH589) induced apoptosis in patients with HCC [37]. Since some patients present drug resistance, exploring other effective therapeutic approaches for hepatic carcinoma is still a great challenge [38]. Thus, treating liver cancer patients by promoting disulfidptosis is a potential way to improve patient survival.

It must be noted out that our research has certain limitations. First, the data used to construct and validate the model were derived solely from public databases, which may not fully reflect the characteristics and conditions of all HCC patients. Second, we selected 16 genes involved in the mechanism of disulfidptosis, of which only a portion have been validated, while some are potential mediators of disulfidptosis. As the investigation into the mechanism of disulfidptosis progresses, the effectiveness of the model we constructed can be further confirmed. Third, it demands more robust assays to explore the molecular mechanisms by which LCAT induces disulfidptosis, and to demonstrate that LCAT is capable of hindering liver cancer metastasis. Moreover, other hub genes of disulfidptosis besides LCAT deserve additional in-depth study. Overall, the DRG score model links disulfidptosis to hepatocellular carcinoma, and could be used for survival prognosis prediction in HCC patients; it provides valuable assistance for personalized treatment approaches.

### Supplementary Information

**Additional file 1: Figure S1.** Consensus cluster analysis of HCC patients based on disulfidptosis-regulated genes. **A** and** B** The cumulative distribution function (CDF) and relative change in the area under the CDF curve when k takes different values. **C**–**F** The diagrams show consistent clustering results when k was 2, 3, 4, and 5. It was most reliable when k was 2. **Figure S2.** The GSEA results of Hallmark pathway and KEGG pathway and GO enrichment analysis base on two DRGs clusters. **Figure S3.** Distribution of hub DRGs at the single-cell level. (**A** and **B**) UMAP plots of GSE202642 and different cell clusters were identified and visualized. **C** Expression of the DRG signature in 9 cell types. **Figure S4.** Biological function analysis of liver cancer cells overexpressing LCAT. **A** Related LCAT expression in liver cancer cells and normal liver cells. **B** Related LCAT expression in LM3 cells that overexpressed LCAT. **C** Related LCAT expression in overexpression LCAT Hep3B cells. **D** and **E** The CCK-8 assay was performed to assess the viability of LM3 and Hep3B cells overexpressing LCAT. **F** and **G** A plate colony formation assay was performed in LM3 and Hep3B cells that were transfected with LCAT.

**Additional file 2: Table S1.** The details of selected 16 disulfidptosis-related genes. **Table S2.** Primers for RT-PCR **Table S3.** 373 differential expression genes between two disulfidptosis-related clusters. **Table S4.** 190 differential expression genes with prognostic significance. **Table S5.** The correlations between the DRGs scores and the sensitivity of 198 drugs in HCC patients.

## Data Availability

Please contact the corresponding author for all data requests.
