# Supplementary figures and images for: Disulfidptosis-related signature predicts prognosis and characterizes the immune microenvironment in hepatocellular carcinoma
Source: Cancer Cell Int. 2024 Jan 9;24:19. doi: 10.1186/s12935-023-03188-y (PMC10775580; doi:10.1186/s12935-023-03188-y)

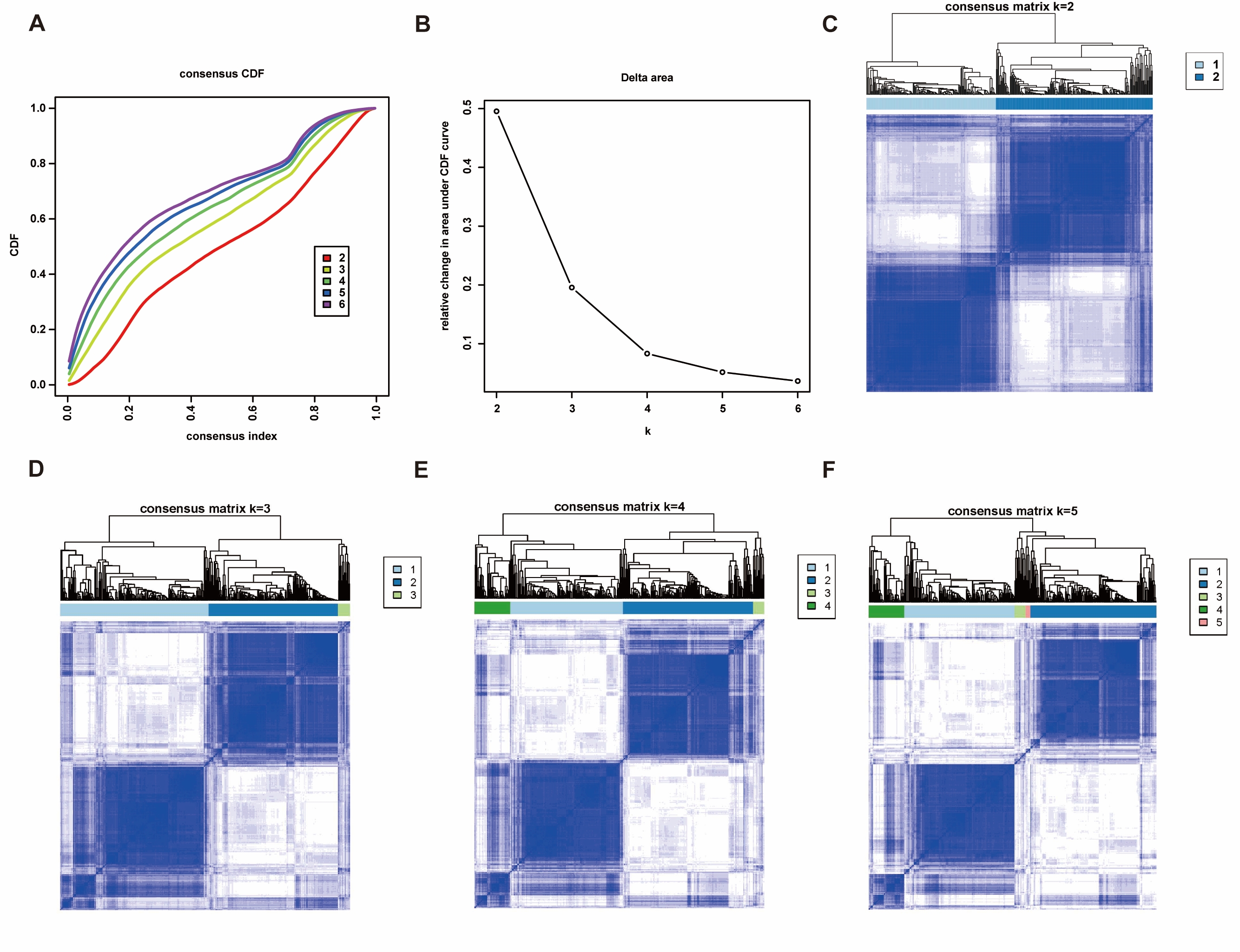

Supplement: Supplementary file 1 — Additional file 1: Figure S1. Consensus cluster analysis of HCC patients based on disulfidptosis-regulated genes. A and B The cumulative distribution function (CDF) and relative change in the area under the CDF curve when k takes different values. C–F The diagrams show consistent clustering results when k was 2, 3, 4, and 5. It was most reliable when k was 2. Figure S2. The GSEA results of Hallmark pathway and KEGG pathway and GO enrichment analysis base on two DRGs clusters. Figure S3. Distribution of hub DRGs at the single-cell level. (A and B) UMAP plots of GSE202642 and different cell clusters were identified and visualized. C Expression of the DRG signature in 9 cell types. Figure S4. Biological function analysis of liver cancer cells overexpressing LCAT. A Related LCAT expression in liver cancer cells and normal liver cells. B Related LCAT expression in LM3 cells that overexpressed LCAT. C Related LCAT expression in overexpression LCAT Hep3B cells. D and E The CCK-8 assay was performed to assess the viability of LM3 and Hep3B cells overexpressing LCAT. F and G A plate colony formation assay was performed in LM3 and Hep3B cells that were transfected with LCAT. [file 12935_2023_3188_MOESM1_ESM.zip › additional/Supplementary Figure 1.jpg]

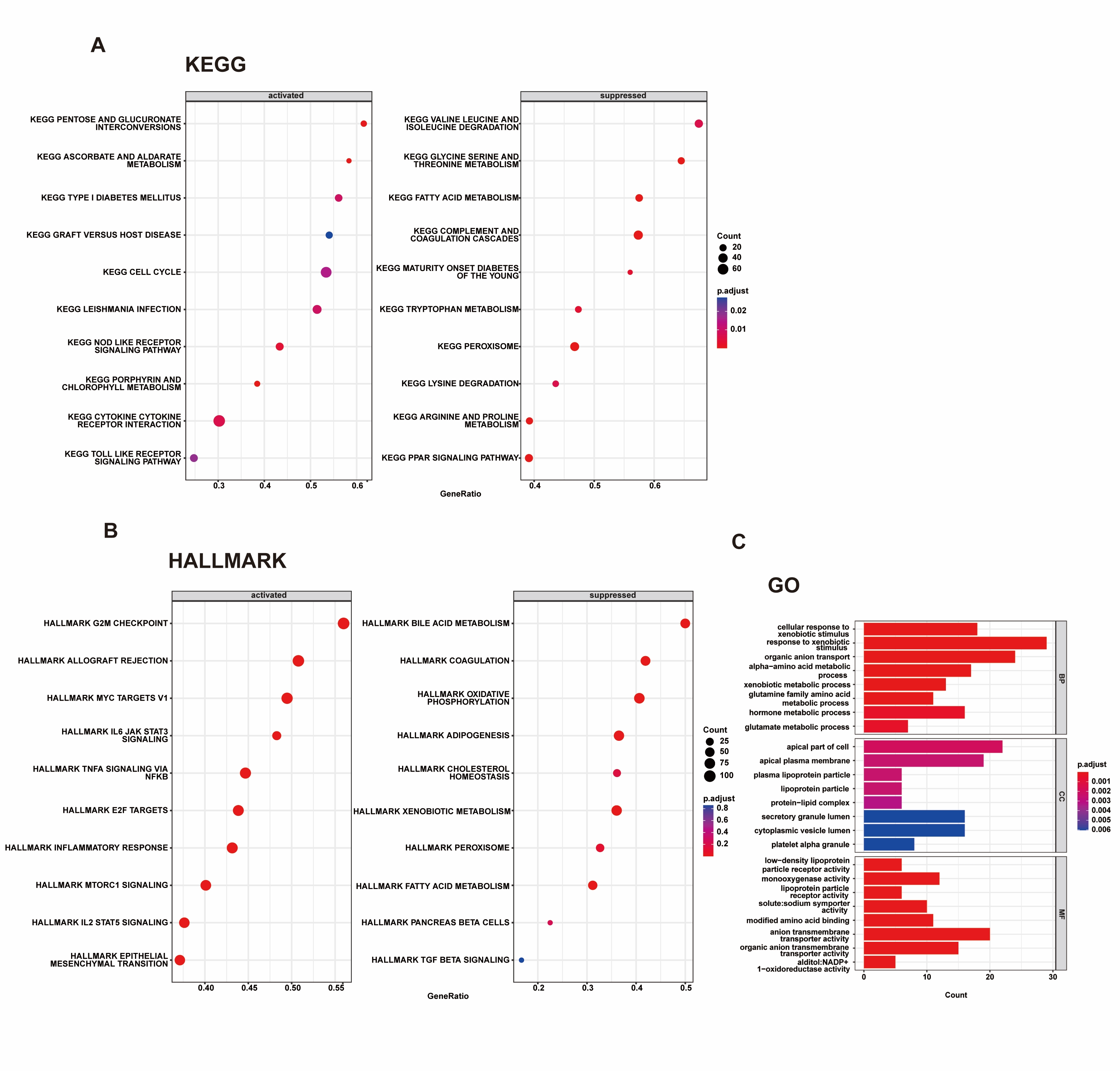

Supplement: Supplementary file 1 — Additional file 1: Figure S1. Consensus cluster analysis of HCC patients based on disulfidptosis-regulated genes. A and B The cumulative distribution function (CDF) and relative change in the area under the CDF curve when k takes different values. C–F The diagrams show consistent clustering results when k was 2, 3, 4, and 5. It was most reliable when k was 2. Figure S2. The GSEA results of Hallmark pathway and KEGG pathway and GO enrichment analysis base on two DRGs clusters. Figure S3. Distribution of hub DRGs at the single-cell level. (A and B) UMAP plots of GSE202642 and different cell clusters were identified and visualized. C Expression of the DRG signature in 9 cell types. Figure S4. Biological function analysis of liver cancer cells overexpressing LCAT. A Related LCAT expression in liver cancer cells and normal liver cells. B Related LCAT expression in LM3 cells that overexpressed LCAT. C Related LCAT expression in overexpression LCAT Hep3B cells. D and E The CCK-8 assay was performed to assess the viability of LM3 and Hep3B cells overexpressing LCAT. F and G A plate colony formation assay was performed in LM3 and Hep3B cells that were transfected with LCAT. [file 12935_2023_3188_MOESM1_ESM.zip › additional/Supplementary Figure 2.jpg]

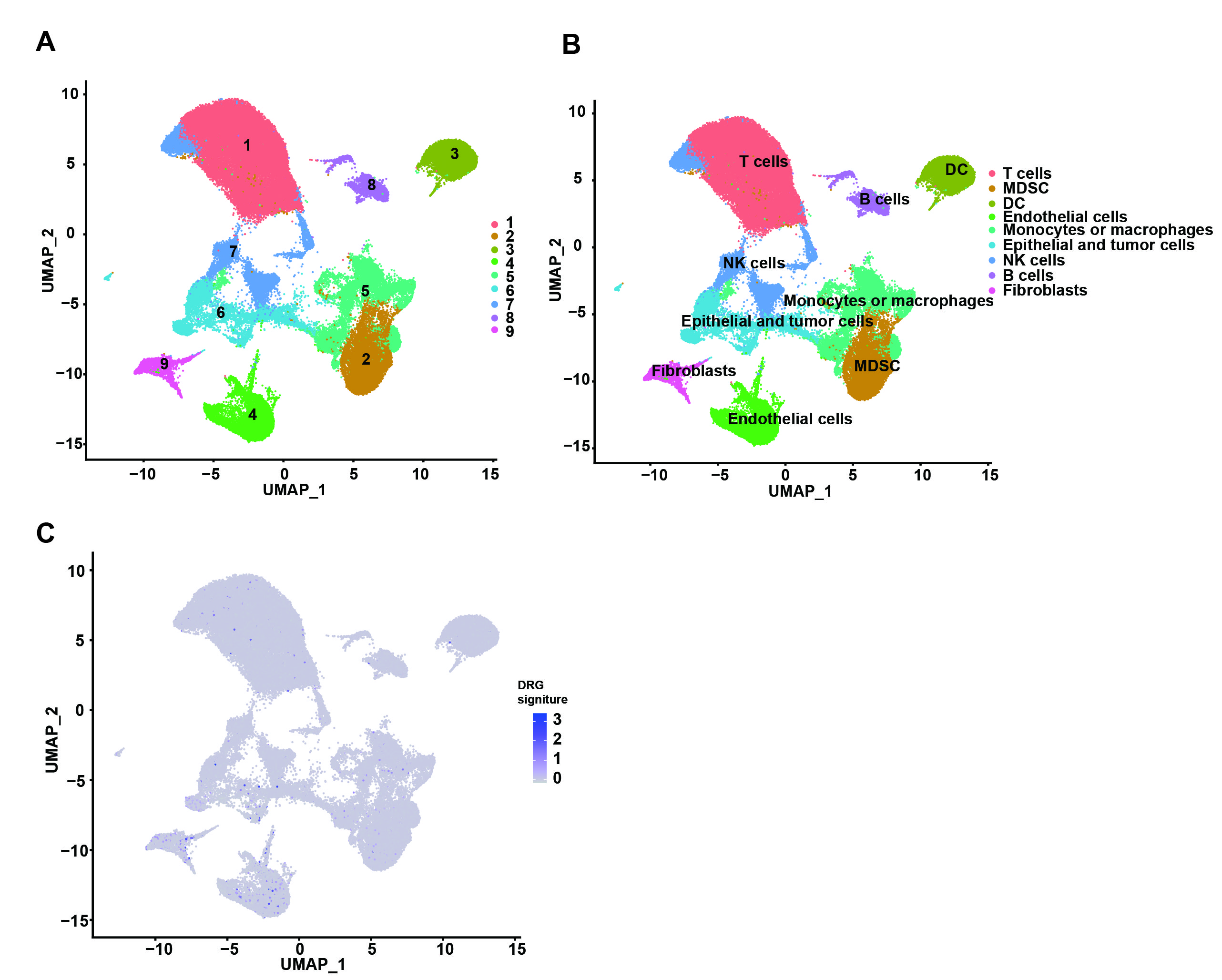

Supplement: Supplementary file 1 — Additional file 1: Figure S1. Consensus cluster analysis of HCC patients based on disulfidptosis-regulated genes. A and B The cumulative distribution function (CDF) and relative change in the area under the CDF curve when k takes different values. C–F The diagrams show consistent clustering results when k was 2, 3, 4, and 5. It was most reliable when k was 2. Figure S2. The GSEA results of Hallmark pathway and KEGG pathway and GO enrichment analysis base on two DRGs clusters. Figure S3. Distribution of hub DRGs at the single-cell level. (A and B) UMAP plots of GSE202642 and different cell clusters were identified and visualized. C Expression of the DRG signature in 9 cell types. Figure S4. Biological function analysis of liver cancer cells overexpressing LCAT. A Related LCAT expression in liver cancer cells and normal liver cells. B Related LCAT expression in LM3 cells that overexpressed LCAT. C Related LCAT expression in overexpression LCAT Hep3B cells. D and E The CCK-8 assay was performed to assess the viability of LM3 and Hep3B cells overexpressing LCAT. F and G A plate colony formation assay was performed in LM3 and Hep3B cells that were transfected with LCAT. [file 12935_2023_3188_MOESM1_ESM.zip › additional/Supplementary Figure 3.jpg]

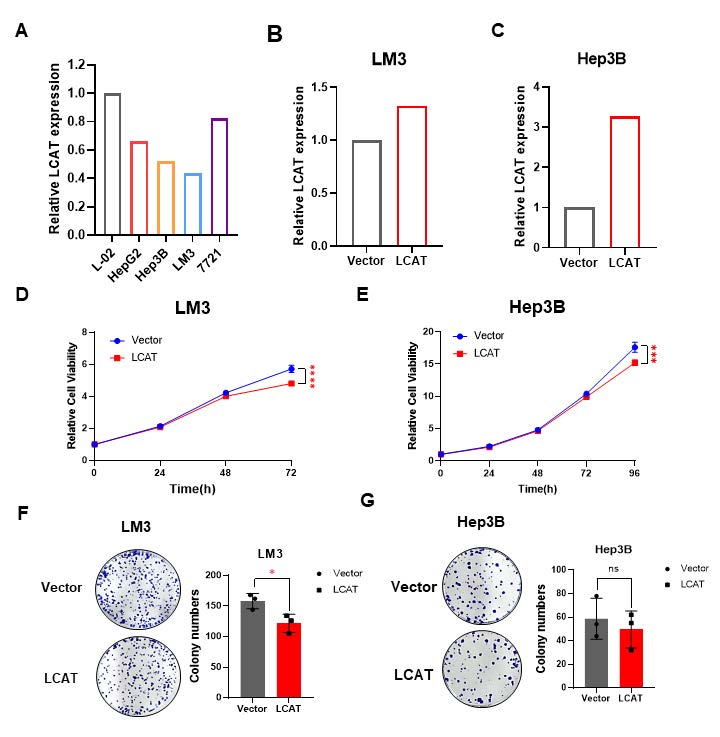

Supplement: Supplementary file 1 — Additional file 1: Figure S1. Consensus cluster analysis of HCC patients based on disulfidptosis-regulated genes. A and B The cumulative distribution function (CDF) and relative change in the area under the CDF curve when k takes different values. C–F The diagrams show consistent clustering results when k was 2, 3, 4, and 5. It was most reliable when k was 2. Figure S2. The GSEA results of Hallmark pathway and KEGG pathway and GO enrichment analysis base on two DRGs clusters. Figure S3. Distribution of hub DRGs at the single-cell level. (A and B) UMAP plots of GSE202642 and different cell clusters were identified and visualized. C Expression of the DRG signature in 9 cell types. Figure S4. Biological function analysis of liver cancer cells overexpressing LCAT. A Related LCAT expression in liver cancer cells and normal liver cells. B Related LCAT expression in LM3 cells that overexpressed LCAT. C Related LCAT expression in overexpression LCAT Hep3B cells. D and E The CCK-8 assay was performed to assess the viability of LM3 and Hep3B cells overexpressing LCAT. F and G A plate colony formation assay was performed in LM3 and Hep3B cells that were transfected with LCAT. [file 12935_2023_3188_MOESM1_ESM.zip › additional/Supplementary Figure 4.jpg]
